# *Alliumalbanicum* (Amaryllidaceae), a new species from Balkans and its relationships with *A.meteoricum* Heldr. & Hausskn. ex Halácsy

**DOI:** 10.3897/phytokeys.119.30790

**Published:** 2019-04-11

**Authors:** Salvatore Brullo, Cristian Brullo, Salvatore Cambria, Giampietro Giusso del Galdo, Cristina Salmeri

**Affiliations:** 1 Department of Biological, Geological and Environmental Sciences, Catania University, Via A. Longo 19, 95125 Catania, Italy Catania University Catania Italy; 2 Department of Biological, Chemical and Pharmaceutical Sciences and Technologies (STEBICEF), Palermo University, Via Archirafi 38, 90123 Palermo, Italy Palermo University Palermo Italy

**Keywords:** *
Allium
*, Amaryllidaceae, Albania, chromosome, new section, taxonomy

## Abstract

A new species, *Alliumalbanicum*, is described and illustrated from Albania (Balkan Peninsula). It grows on serpentines or limestone in open rocky stands with a scattered distribution, mainly in mountain locations. Previously, the populations of this geophyte were attributed to *A.meteoricum* Heldr. & Hausskn. ex Halácsy, described from a few localities of North and Central Greece. These two species indeed show close relationships, chiefly regarding some features of the spathe valves, inflorescence and floral parts. They also share the same diploid chromosome number 2*n* =16 and similar karyotype, while seed testa micro-sculptures and leaf anatomy reveal remarkable differences. There are also several morphological features that allow them to be differentiated at specific level. The inclusion of both species into a newly described section Pseudoscorodon of the subgen. Allium is proposed. An analytic key to the species, included in the new section, is also provided.

## Introduction

One of the richest and largest genera of Monocotyledons is *Allium* L. and it is almost exclusively widespread in the northern hemisphere, where it is represented by ca. 1200 taxa (Govaerts et al. 2018). This genus is characterised by a high rate of endemism, especially observable in North America, Asia and the Mediterranean area, which represent the main centres of diversity.

In the context of cytotaxonomical research on the genus *Allium* in the Mediterranean territories, especially regarding the subgen. Allium (Bogdanović et al. 2008, 2009, 2011a, 2011b, Brullo et al. 1997a, 1997b, 1999, 2001, 2003a, 2003b, 2004, 2007, 2008a, 2008b, 2009, 2010, 2014, 2017, 2018, Özhatay et al. 2018), a peculiar population occurring in Albania, previously attributed to *A.meteoricum* Heldr. & Hausskn. ex Halácsy, is examined. *Alliummeteoricum* s. str. was described from Meteora in Central Greece by Halácsy (1904) and later also recorded from Assopos, Greece (Tzanoudakis 1983, Tzanoudakis and Vosa 1988, Brullo et al. 2001). Extensive morphological investigations, carried out on herbarium material and living specimens coming from Albania (Devoli river near Berat on serpentines) and Greece (Meteora on sandstones), allowed us to verify that the Albanian populations are very different from those of Meteora, which is the locus classicus of *A.meteoricum*. Detailed analyses regarding the chromosome complement and karyotype structure, seed testa micro-morphology and leaf anatomy provided relevant discriminant features. Based on these data, the Albanian populations were referred to a species new to science, named *Alliumalbanicum*.

## Materials and methods

Plant morphology was analysed on 20 living mature plants. Qualitative and quantitative morphological characters, considered as diagnostic in *Allium*, were analysed and scored (Table 1) on fresh material. Comparison of *A.albanicum* with *A.meteoricum* was based on living plants coming from the type locality of both species, collected by S. Cambria in Albania (June 2017) and by S. Brullo and C. Cambria in Greece (June 2018), as well as on several herbarium specimens (BM, CAT, G, K, W, WU) in order to check the correct sample identification. Literature data were also considered. Collected specimens are preserved in CAT.

For the karyological study, living bulbs were collected and potted at the Botanical Garden of Catania University. Root tips were pre-treated with 0.3% (w/v) colchicine water solution for 3 h at room temperature and then fixed overnight in fresh Farmer’s fixative (3:1 v/v, absolute ethanol: glacial acetic acid). Root tips were hydrolysed in 1N HCl at 60 °C for 7 min, washed and stained with Feulgen for 1 h. Microphotographs of good quality metaphase plates were taken with a Zeiss Axioskop2 light microscope equipped with an Axiocam MRc5 high resolution digital camera. Chromosome number and karyotype details were analysed from 10 well spread metaphase plates from 5 individuals, the mean values being used for the karyotype characterisation. Metaphase chromosomes were measured using the image analysis system Zeiss Axiovision 4.8, while karyotyping was performed by CROMOLAB 1.1 software Brullo (2002). The chromosome types were named according to the position of the centromere: r = 1–1.3 (m) median, r = 1.3–1.7 (msm) median-submedian, r = 1.7–3 (sm), r = 3–7 (st) subterminal (Tzanoudakis 1983). All measured karyomorphometric parameters are given in Table 2. Karyotype symmetry indices followed Paszko (2006) and Peruzzi and Eroğlu (2013).

Leaf anatomy was studied on living materials coming from the type locality and cultivated in the Botanical Garden of Catania University. Leaf blades of maximum size, in their optimal vegetative development, usually before the flowering stage, were taken from the middle part and fixed in Carnoy. Leaf cross sections were double stained with ruthenium red and light green, analysed and photographed with a light microscope (Zeiss Axioskop2 and Axiocam MRc5 digital camera).

Seed testa micro-morphology was analysed on mature and dry material taken from individuals coming from the type locality, using a scanning electron microscope (SEM) Zeiss EVO LS10, according to the protocol reported by Stork et al. (1980). Terminology of the seed coat sculpturing follows Barthlott (1981, 1984) and Gontcharova et al. (2009).

**Table 1.** Main diacritic features of *Alliumalbanicum* and *A.meteoricum*.

Characters	* A. albanicum *	* A. meteoricum *
Bulb size (mm)	8–10 × 5–10	10–14 × 8–12
Bulb outer coat colour	brownish	blackish-brown
Stem height (cm)	14–28(-30)	10–25
Stem diameter (mm)	1	1–1.2
Stem coverage by leaf sheaths	1/4	1/2
Leaf number	3	3–4
Leaf length (cm)	up to 10	up to 12
Spathe valves length (mm)	subequal, 8–12	unequal, 7–11
Spathe valve appendage length (mm)	1–2.5	1–4
Spathe valves arrangement	fused up to 1/2	free
Larger spathe valve nerves (no.)	3–5	5
Smaller spathe valve nerves (no.)	3	3–5
Pedicel length (mm)	6–25	6–15
Tepal colour	white tinged with pink	purplish-pink
Tepal midvein colour	greenish-purple	purplish
Tepal length (mm)	5.5–6.5	6–7.5
Tepal apex	eroded	rounded
Stamen filament colour	yellowish above, white below	white
Outer stamen filament length (mm)	1.7–2.1	2.7–3.3
Inner stamen filament length (mm)	2.5–3.2	3.5–4
Anther colour	greenish-pale yellow	yellow
Anther apex	rounded	apiculate
Annulus height (mm)	0.5–0.6	0.7–0.9
Ovary colour	yellow	green
Ovary apex	slightly wrinkled	smooth
Ovary nectariferous pores height	about 1/2 ovary	about 1/4 ovary
Style length (mm)	2.7–2.8	1.5–2
Capsule length (mm)	4–4.5	3–3.5
Capsule shape	subglobose-obovate	subglobose
Seed size (mm)	3.5–4.0 × 2.4–2.5	2.2–2.5 × 1.9–2.0

**Table 2.** Karyomorphometric parameters and symmetry indices for *Alliumalbanicum* and *A.meteoricum*. Mean values were calculated from 10 good metaphase plates from individuals of the type locality.

*** Allium albanicum ***								
**Pairs**	**LA (µm)**	**SA (µm)**	**TAL (µm)**	**TRL**%	**AR**	**CI**	**CA**	**Type**
I	5.00 ± 1.43	3.74 ± 1.07	8.74 ± 2.48	7.87 ± 0.59	1.34	42.82	0.14	msm
II	4.52 ± 0.94	3.38 ± 1.05	7.91 ± 1.71	7.16 ± 0.34	1.34	42.80	0.14	msm
III	4.03 ± 0.87	3.48 ± 0.73	7.53 ± 0.58	6.84 ± 0.34	1.16	46.20	0.07	m
IV	4.67 ± 0.99	1.99 ± 0.38	6.79 ± 1.31	6.19 ± 0.27	2.35	29.30	0.40	sm^sat^
V	3.56 ± 0.40	3.09 ± 0.34	6.78 ± 0.66	6.26 ± 0.59	1.15	45.57	0.07	m^sat^
VI	3.48 ± 0.77	2.93 ± 0.61	6.44 ± 1.34	5.86 ± 0.42	1.19	45.56	0.09	m
VII	3.33 ± 0.75	2.10 ± 0.38	5.43 ± 0.38	4.91 ± 0.81	1.58	38.69	0.23	msm^sat^
VIII	2.87 ± 0.42	2.33 ± 0.42	5.33 ± 0.77	4.90 ± 0.47	1.23	43.68	0.10	m^sat^
**TCL**: 109.88 ± 21.7 µm; **MCL**: 6.87 ± 1.2 µm; **d-value**: 16.83; **DRL**%: 3.28; **S**%: 57.95;**MAR**: 1.37; **MCI**: 41.83; **Cv_CL_**: 17.12; **Cv_CI_**: 13.00; **M_CA_**: 15.63								
*** Allium meteoricum ***								
**Pairs**	**LA (µm)**	**SA (µm)**	**TAL (µm)**	**TRL**%	**AR**	**CI**	**CA**	**Type**
I	4.05 ± 0.21	3.23 ± 0.30	7.27 ± 0.31	7.78 ± 0.02	1.26	44.35	0.11	m
II	3.87 ± 0.44	3.06 ± 0.73	6.94 ± 1.01	7.42 ± 0.35	1.26	44.19	0.12	m
III	3.79 ± 0.43	2.74 ± 0.23	6.53 ± 0.34	6.99 ± 0.37	1.38	41.98	0.16	msm
IV	4.15 ± 0.16	1.69 ± 0.11	6.16 ± 0.27	6.59 ± 0.29	2.45	27.49	0.42	sm^sat^
V	2.90 ± 0.35	2.58 ± 0.11	5.48 ± 0.11	5.87 ± 0.12	1.13	47.06	0.06	m^sat^
VI	2.90 ± 0.23	2.50 ± 0.34	5.40 ± 0.57	5.78 ± 0.61	1.16	46.27	0.07	m
VII	2.66 ± 0.11	1.85 ± 0.11	4.52 ± 0.23	4.83 ± 0.24	1.43	41.07	0.18	msm
VIII	2.42 ± 0.23	2.02 ± 0.42	4.44 ± 0.57	4.74 ± 0.51	1.20	45.45	0.09	m
**TCL**: 93.48 ± 21.7 µm; **MCL**: 5.84 ± 1.06 µm; **d-value**: 14.13; **DRL**%: 3.5; **S**%: 55.31;**MAR**: 1.36; **MCI**: 42.21; **Cv_CL_**: 18.16; **Cv_CI_**: 14.48; **M_CA_**: 15.21								

**Abbreviations**: LA = long arm; SA = short arm; TAL = total absolute length; TRL = total relative length; AR = arm ratio; CI = centromeric index; CA = centromeric asymmetry; Type=chromosome nomenclature; sat = satellited; TCL = total chromosome length; MCL = mean chromosome length; d-value = difference between Long arms and Short arms; DRL% = difference of relative length; S% = Relative length of shortest chromosome; MAR = mean arm ratio; MCI = mean centromeric index; Cv_CL_ = coefficient of variation of chromosome length; Cv_CI_ = coefficient of variation of centromeric index; MCA = mean centromeric asymmetry.

## Taxonomy

### Allium albanicum

Taxon classificationPlantaeAsparagalesAmaryllidaceae

Brullo, C. Brullo, Cambria, Giusso & Salmeri sp. nov.

urn:lsid:ipni.org:names:60478500-2

Figs 1 , 7B–D

Allium meteoricum auct. fl. Albania non Halacsy, Consp. Fl. Graec. 3(1): 250. 1904, **Syn.**

#### Type.

ALBANIA. Devoli river, near Berat, serpentines, ca. 700 m elev., 40°43'12.00"N, 20°32'18.00"E, 26 June 2017, *S. Cambria s.n.* (Holotype: CAT; Isotypes: CAT, FI, G).

#### Diagnosis.

Allio meteoricum similis sed bulbis minoribus tunicis exterioribus brunneis, scapo ad 1/4 longitudinem vaginis foliorum tecto, spathae valvis in dimidio inferiore connatis, appendice usque ad 2,5 mm longa, majore 3–5 nervata, minore 3 nervata, tepalis albo-roseis, minoribus, apice erosis, filamentis staminorum minoribus, luteis superne, annulo breviore, antheris viridulis- pallide luteis, apice rotundatis, ovario luteo leviter apice rugoso, poris nectariferis majoris, capsula majore subgloboso-obovata, differt.

#### Description.

Bulb ovoid, 8–10 × 5–10 mm, with outer tunics coriaceous, brownish, the inner membranous, whitish. Stem 14–28(30) cm tall, cylindrical, flexuous, 1–1.5 mm in diameter, glabrous, erect, covered for 1/4 of its length by the leaf sheaths. Leaves 3, rather flat, glabrous, green, ribbed, up to 10 cm long and 1–2.2 mm wide, denticulate at margins. Spathe persistent, with 2 valves subequal, 8–12 mm long, shorter than the inflorescence, fused to half of their length, with an appendage 1–2.5 mm long, the larger 3–5-nerved, the smaller 3-nerved. Bostryces 12. Inflorescence laxly hemispheric, 2–3 cm in diameter, many flowered, with unequal pedicels 6–25 mm long. Perigon cylindrical-urceolate, with tepals of equal length, white tinged with pink, mid-vein greenish-purple, the inner ones linear-elliptical, the outer ones sublanceolate, rounded and slightly eroded at the apex, 5.5–6.5 mm long and 1.7–2 mm wide. Stamens included, with simple filament yellowish above and whitish below, the outers 1.7–2.1 mm long, the inners 2.5–3.2 mm long, below connate into an annulus 0.5–0.6 mm high. Anthers greenish-pale yellow, elliptical, 1–1.1 × 0.6 mm, rounded at the apex. Ovary subglobose-ovoid, yellow, slightly wrinkled at the apex, 1.5–1.7 × 1.4–1.7 mm, with large nectariferous pores, long about half the ovary. Style white, 2.7–2.8 mm long, stigma capitate. Caspule trivalved, subglobose-obovate, 4–4.5 mm, with evident nectariferous pores.

**Figure 1.**
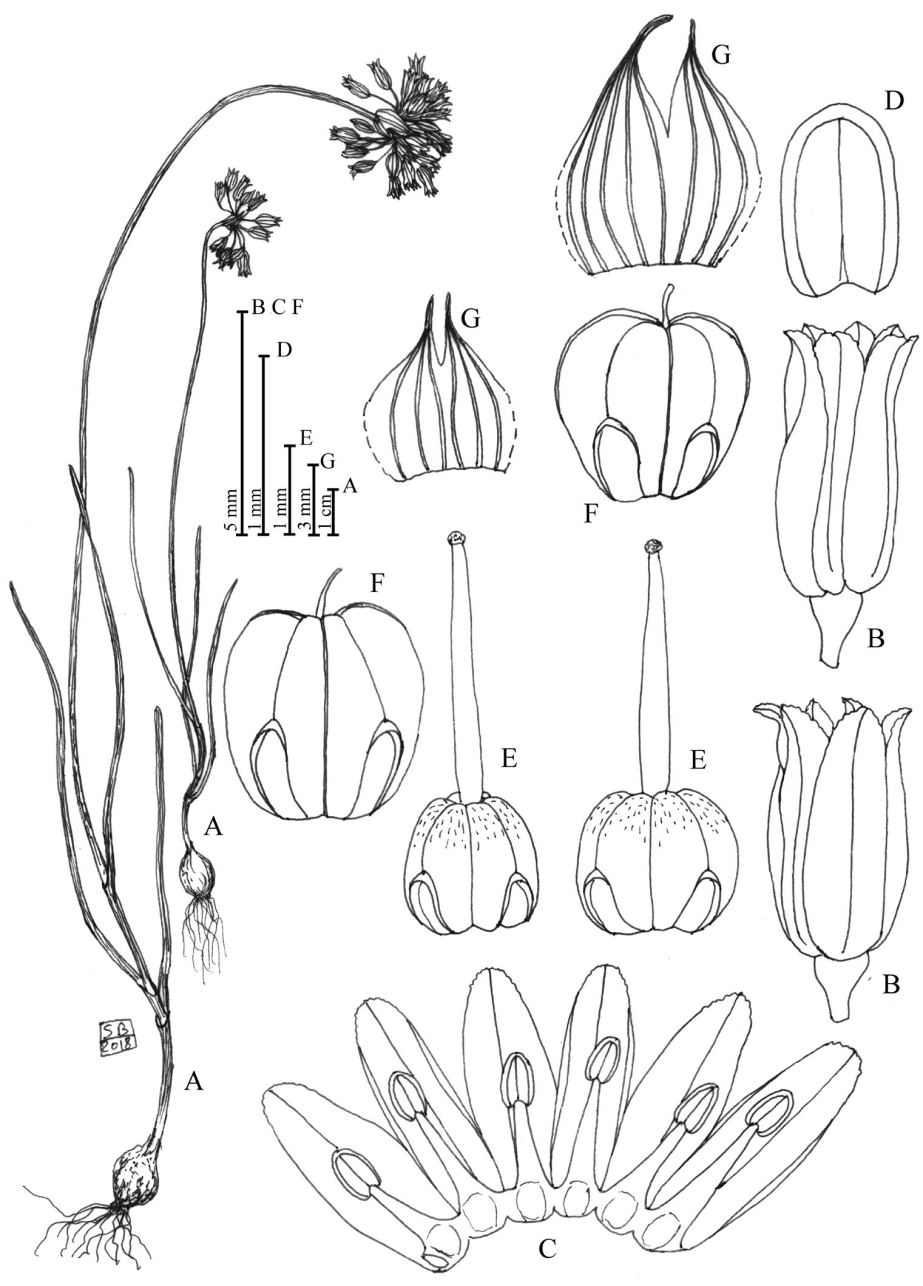
*Alliumalbanicum* Brullo, C. Brullo, Cambria, Giusso & Salmeri sp. nov. **A** Habit **B** Flower **C** Perigon and stamens open **D** Anther **E** Ovaries **F** Capsule **G** Spathe valves. Drawing by S. Brullo based on living material coming from the type locality.

#### Phenology.

Flowering and fruiting from June to July.

#### Etymology.

The epithet refers to the Latin “*Albanicum*”, coming from Albania, the country where the species grows.

#### Karyology.

**Figure 2.**
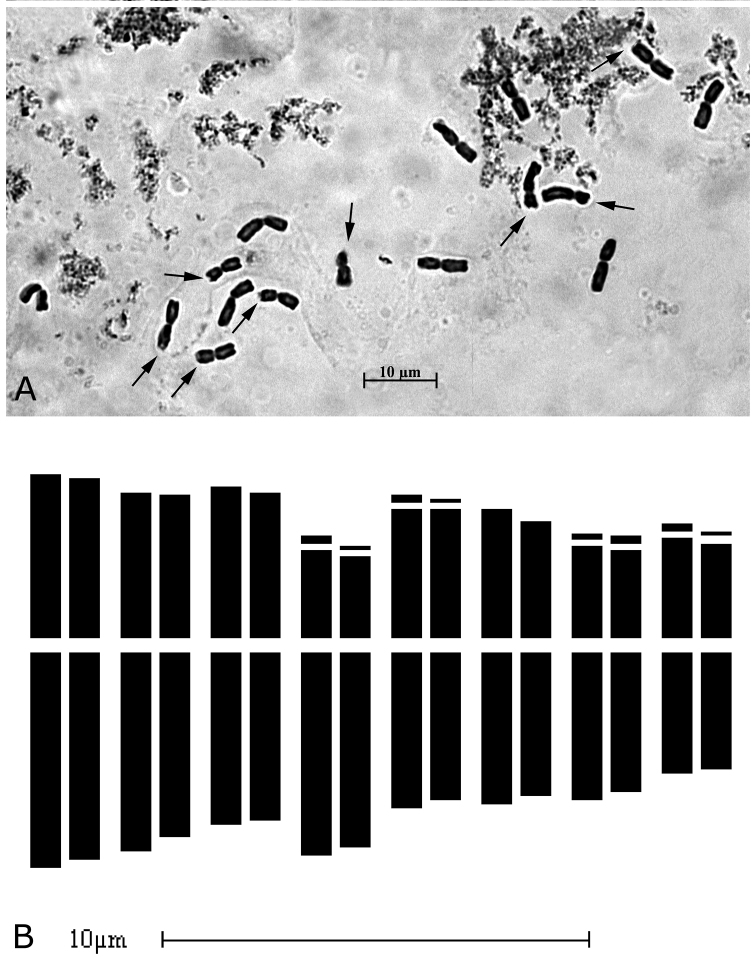
Chromosome complement (2*n* = 2*x* = 16) of *Alliumalbanicum*. **A** Mitotic metaphase plate from type locality; arrows indicate satellited chromosomes **B** idiogram.

The investigated specimens of *A.albanicum* from the type locality revealed a diploid chromosome number with 2*n* = 16. The karyotype obtained from somatic metaphase plates (Fig. 2A) is mostly characterised by nearly metacentric chromosomes; specifically, the mean karyogram (Fig. 2B) reveals 4 typical metacentric (m) pairs (III, V, VI, VIII), 3 meta- submetamentric (msm) pairs (I, II, VII), having an arm ratio between 1.30 and 1.67 and one submetacentric (sm) pair (IV). Microsatellites were detected on the short arms of two metacentric chromosome pairs, one meta-submetacentric pair and the submetacentric one. Thus, the chromosome formula can be expressed as 2*n* = 2*x* = 16: 4 m + 4 m^sat^ + 4 msm + 2 msm^sat^ + 2 sm^sat^. Chromosomes have a total length varying from 8.90 ± 2.5 µm of the longest chromosome to 5.16 ± 0.8 µm of the shortest one, while the relative length ranges from 8.01% to 4.73%. As already emphasised by Tzanoudakis (1983) and Brullo et al. (2001), *A.meteoricum* also has a diploid chromosome complement with 2*n* = 16 (cf. Brullo et al. 2001, Fig. 6A), which is characterised by 5 metacentric chromosome pairs, two of which microsatellited on the short arm, 2 msm pairs and one submetacentric microsatellited pair (cf. Brullo et al. 2001, fig. 8A). Chromosomes vary in total length from 7.29 µm of the longest chromosome to 4.03 µm of the shortest one, while the relative chromosome length ranges from 7.8% to 4.3%. Table 2 shows the mean values for all measured karyomorphometric parameters and symmetry indices of *A.albanicum* and *A.meteoricum* from the type locality.

#### Leaf anatomy.

The leaf cross section of *A.albanicum* shows a flat outline, with some dorsal ribs. The epidermis is formed by small cells covered by a well-developed cuticle externally more thickened. Stomata are numerous and distributed along the whole leaf perimeter. The palisade tissue is regular and compact, arranged in one layer of long cylindrical cells, more developed on the adaxial face. The spongy tissue is rather compact and slightly lacunose, in the peripheral part many secretory canals occur. The maximum number of vascular bundles is 20, 11 of which are very small and are localised on the adaxial face, while on the abaxial face, there is one large central vascular bundle and 4 smaller ones for each side (Fig. 4).

**Figure 3.**
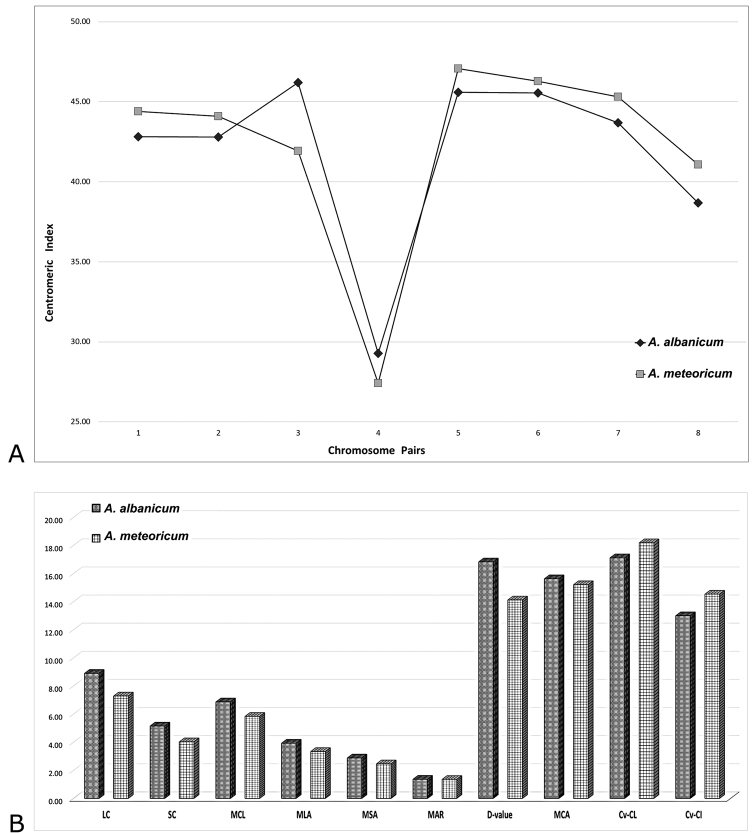
Comparison of karyotype morphometric data between *Alliumalbanicum* and *A.meteoricum*. **A** Variation of centromeric index for each chromosome pair **B** Variation of the main karyomorphometric parameters and symmetry indices (*LC* longest chrom., *SC* shortest chrom.; *MCL* mean chromosome length; *MLA* mean long arm; *MSA* mean short arm; other abbreviations see Table 2).

**Figure 4.**
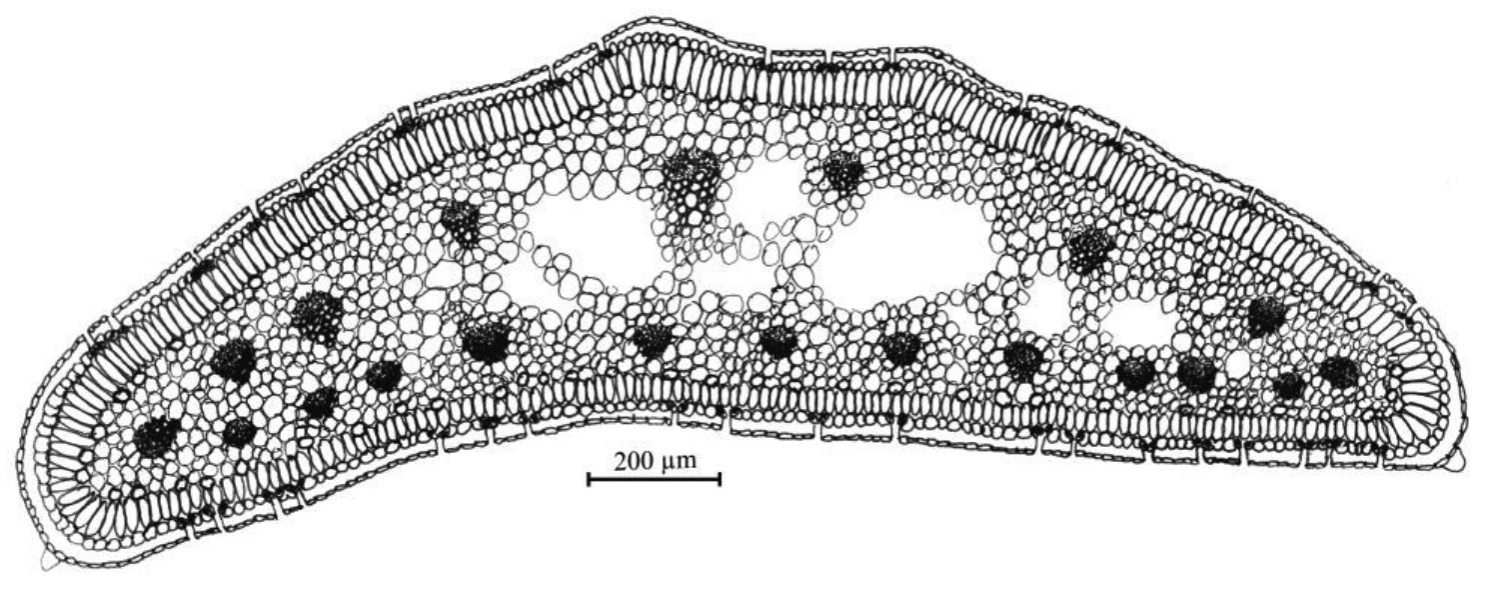
Leaf cross section of *Alliumalbanicum* from living material coming from the type locality. Drawing by S. Brullo.

#### Seed micromorphology.

**Figure 5.**
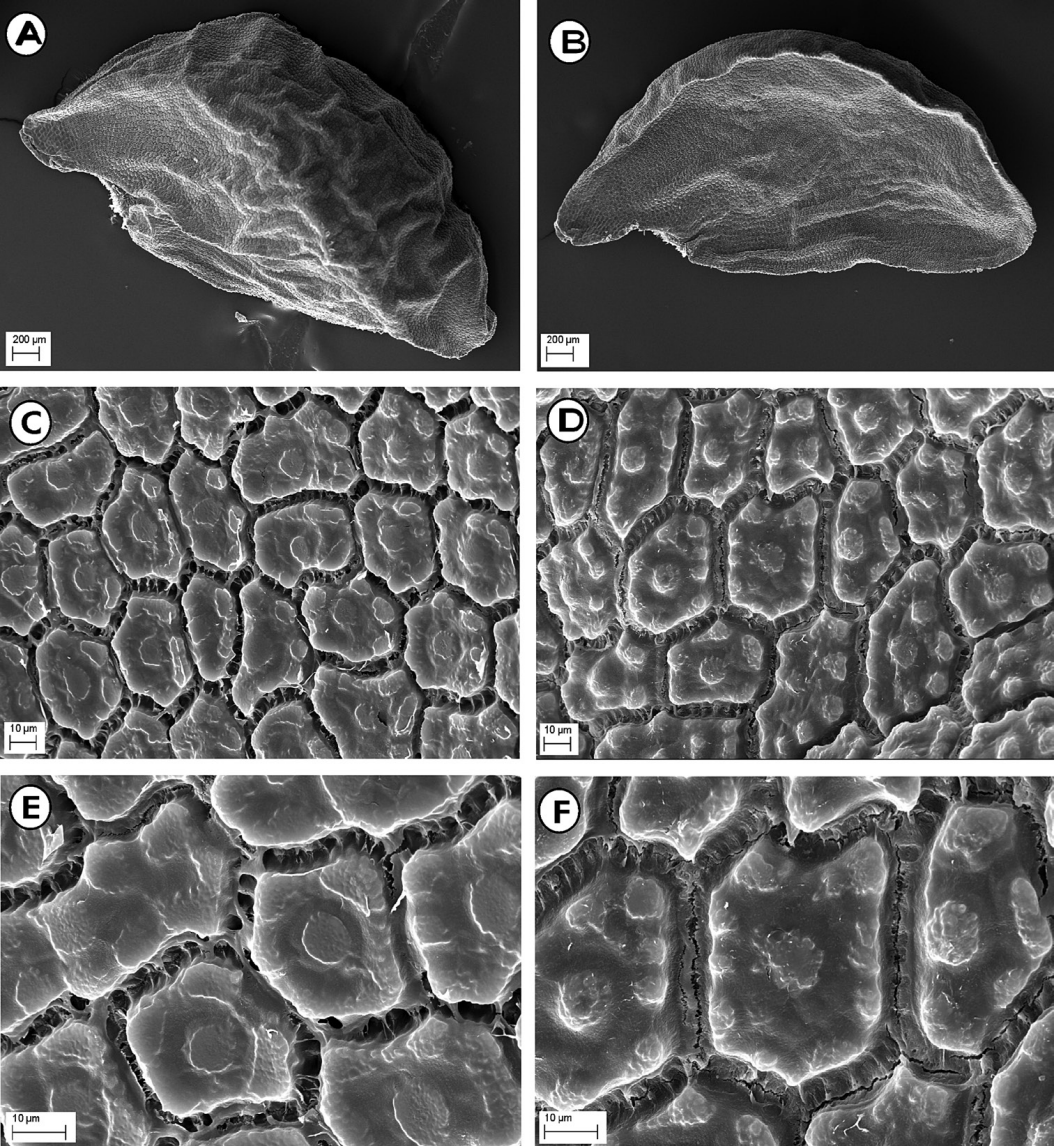
SEM micrographs of the seed coat of *Alliumalbanicum*. **A** Seed (dorsal face, 30×) **B** Seed (ventral face, 30×) **C** Seed coat (central part of dorsal face, 600×) **D** Seed coat (central part of ventral face, 600×) **E** Seed coat (central part of dorsal face, 1200×) **F** Seed (central part of ventral face, 1200×). Photos from material of type locality (CAT).

**Figure 6.**
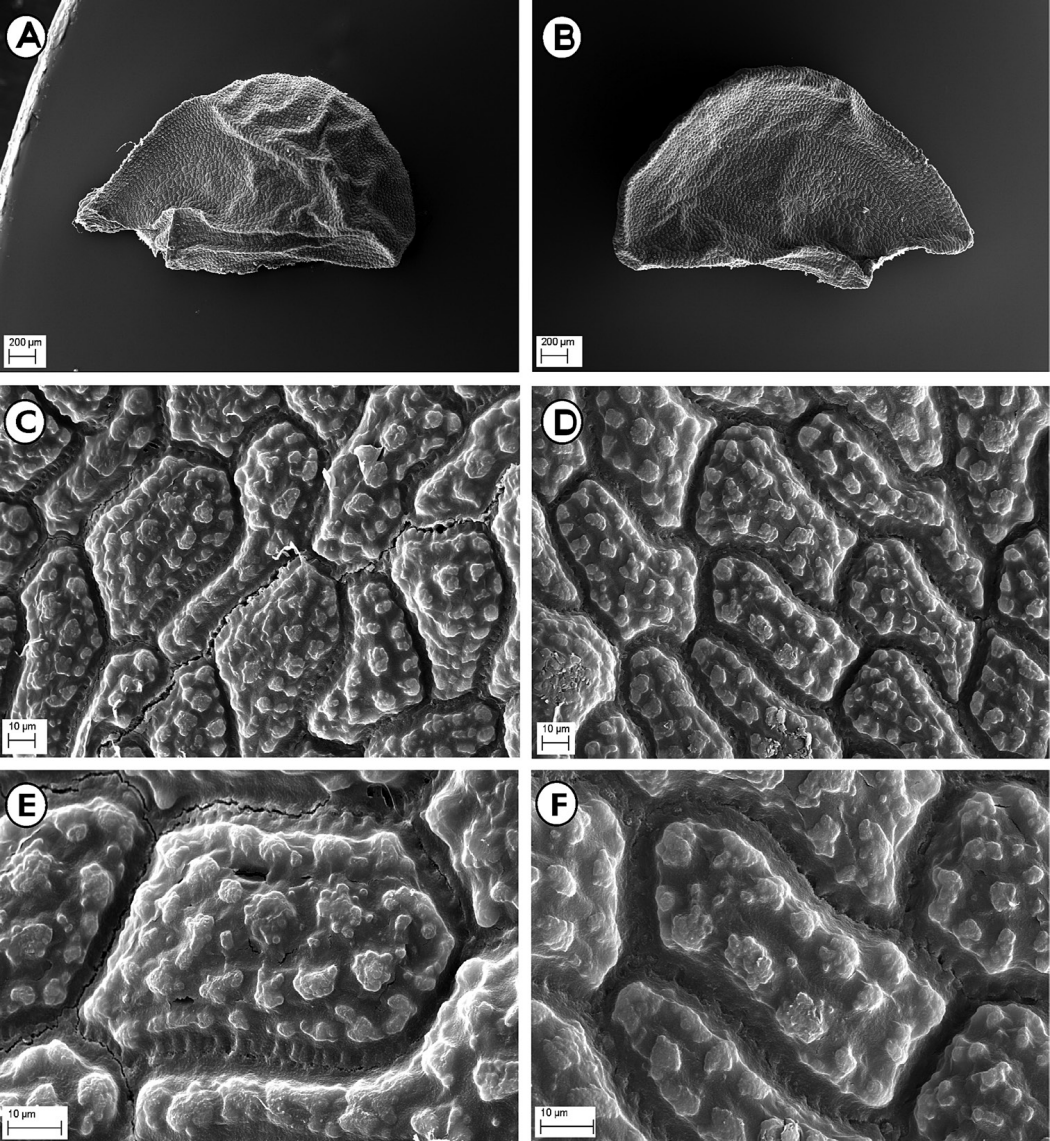
SEM micrographs of the seed coat of *Alliummeteoricum*. **A** Seed (dorsal face, 30×) **B** Seed (ventral face, 30×) **C** Seed coat (central part of dorsal face, 600×) **D** Seed coat (central part of ventral face, 600×) **E** Seed coat (central part of dorsal face, 1200×) **F** Seed (central part of ventral face, 1200×). Photos from material of type locality (CAT).

As emphasised by numerous authors (Pastor 1981, Češmedžiev and Terzijski 1997, Fritsch et al. 2006a, Neshati and Fritsch 2009, Celep et al. 2012, Salmeri et al. 2016, Lin and Tan 2017, Özhatay et al. 2018, Brullo et al. 2018), the micro-sculptures of the seed testa in the *Allium* species represent a very stable and conservative character, showing usually relevant taxonomical and phylogenetical implications. Seeds of *A.albanicum* at low magnification (30×) showed a semi-ovoid shape (3.5–4.0 × 2.4–2.5 mm), with a rather rugose surface (Fig. 5A, B). The seeds observed at high magnification (600× and 1200×) revealed irregularly polygonal testa cells, having a size of 40–80 × 17–40 µm (Fig. 5C–F). The anticlinal walls appeared flat, rather straight and partly covered by strip-like sculptures forming a widened intercellular region, not or just a little lacerate. The periclinal walls were flat, with few flat and smooth or slightly knobby verrucae, usually arranged along the margin surrounding a central one. Conversely, the seeds of *A.meteoricum* at low magnification (30×) revealed a semi-globose shape and a smaller size (2.2–2.5 × 1.9–2.0 mm), with less pronounced surface roughness (Fig. 6A, B). The seeds observed at high magnification (600× and 1200×) also showed irregularly polygonal testa cells, but with a larger size (60–120 × 15–50 µm) (Fig. 6C–F). The anticlinal walls appeared flat, rather straight and partly covered by strip-like sculptures forming a widened intercellular region, partially lacerate. The periclinal walls were weakly protruding with several knobby verrucae distributed over the whole surface.

#### Ecology and distribution.

**Figure 7.**
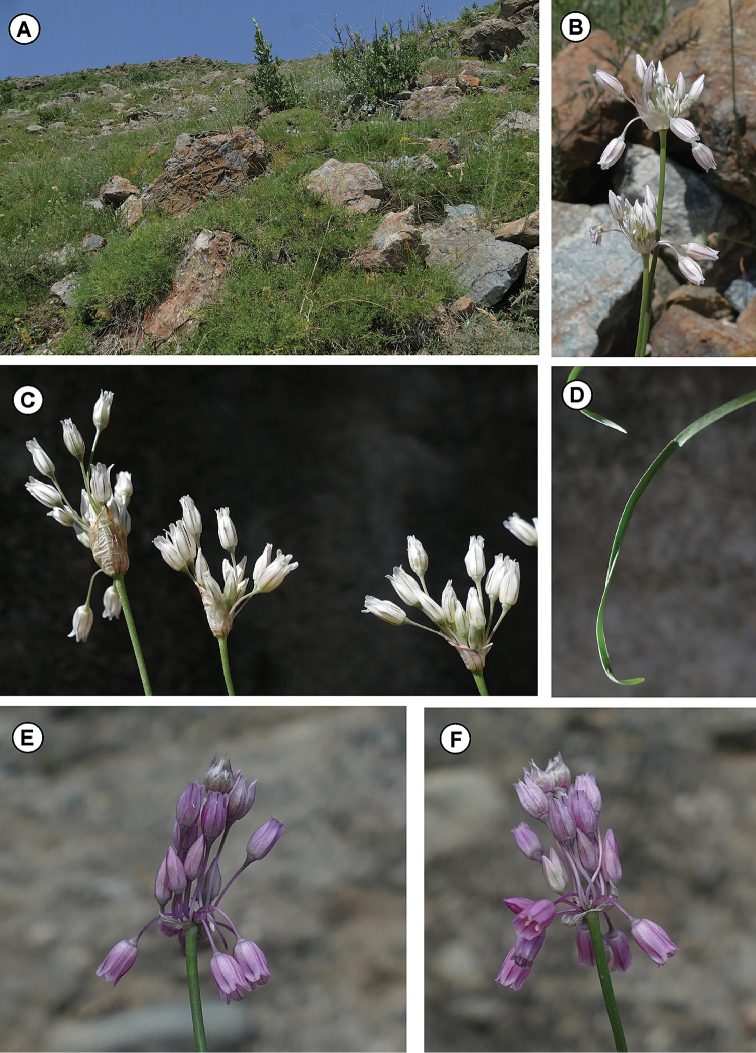
Phenological features of *Alliumalbanicum* and *A.meteoricum*. **A** Growing habitat of *A.albanicum* in the locus classicus (Albania) **B** Individuals of *A.albanicum* from the locus classicus **C***A.albanicum* cultivated material in Botanical Garden of Catania **D** Leaf of *A.albanicum*, cultivated material **E, F** Individual of *A.meteoricum*, from Meteora (Greece). Photos by S. Cambria.

The investigated population of *A.albanicum*, previously reported as sub *A.meteoricum* (Pils 2016, Barina 2017), was collected on serpentinic substrata of open stands characterised by rocky outcrops at ca. 700 m of elevation (Fig. 7). Plants grow in shrub vegetation differentiated by some serpentinicolous plants, such as *Acantholimonalbanicum* O.Schwarz & F.K.Mey, *Centaureasalonitana* Vis., *Centranthuslongiflorus* Steven, *Festucopsisserpentini* (C.E. Hubb.) Melderis, *Forsythiaeuropaea* Degen & Bald., *Iberisumbellata* L., *Salviaringens* Sibth. & Sm. etc. According to literature (Pils 2016, Barina 2017) and herbarium investigations, *A.albanicum* seems to have a scattered distribution in Albania, though its effective geographic range might be better defined only through further field surveys. Based on Brullo et al. (2001), Dimopoulos et al. (2013) and personal herbarium surveys, *A.meteoricum* is a Greek endemic, circumscribed to northern and central Greece and further populations reported in other Greek sites or different territories cannot be referred to this species. Therefore, the remaining Albanian populations referred to as *A.meteoricum* should also be checked in detail as regards their taxonomic attribution.

#### Additional specimens examined.

ALBANIA. In humidis collinis serpentinum ad Renci distr. Scutari, 11 June 1897, *Baldacci 85a* (BM, G); In humidis collinis serpentinii ad Renci distr. Scutari, June 1897, *Baldacci 355* (WU); Nordost Albanien, auf Felsen in der subalp. Region des Pastrik ca. 1200 m elev., 31 July 1914, *Dorfler 593* (WU); Nord Albania, Umgebung von Shkodra Abhänge des kleinen Bordans alt. Serpentin, 8 June 1916, *Janchen s. n.* (WU); Hasi Pastrik an Felsen des westlichen Ausläufers, ca. 1200 m elev., 22 July 1918. *Dorfler 908* (BM, K, W, WU).

#### Examined specimens of *Alliummeteoricum*.

GREECE, Thessalia superior in collibus circa monasteria Meteora supra Kalabaka (Aeginium veterum), substrata diluviali e saxis conglomeratis, 15/16 July 1885 *Heldreich s. n*., sub *Alliummeteoricum* Heldreich & Haussknecht sp. nova (WU Herbarium Halacsy); this specimen, already quoted by Brullo et al. 2001 as a type of *A.meteoricum*, is more completely reported and here correctly designated as lectotype of *Alliummeteoricum*. Thessalia, Trikkala at Meteora above Kalambaki, sunny rocks, 29/06/2018, *S. Brullo & S. Cambria s. n.* (CAT); for other examined specimens, see Brullo et al. (2001).

#### Discussion.

For its general habit and some features such as flat leaves, spathe valves very short, 3–5 nerved, briefly appendiculate, umbel laxly subglobose, perigon cylindrical-urceolate, stamens not exserted, ovary with evident nectariferous pores, the populations of *A.albanicum* were previously referred to as *A.meteoricum* (Halacsy 1904, Hayek 1932, Bornmüller 1933, Stearn 1978, 1980, Meyer 2011, Vangjeli 2015, Pils 2016, Barina 2017).

In light of in-depth taxonomical investigations carried out on living and herbarium material, the analysed Albanian populations are well differentiated from those of *A.meteoricum* coming from the locus classicus, formerly studied by Brullo et al. (2001). Table 1 summarises the most relevant morphological characters differentiating the two species, which mainly consist in the different size and colour of bulbs and tepals, length of the scape covered by the leaf sheaths, shape of spathe valves, colour and size of stamens, ovary and capsule and the shape of nectariferous pores. In particular, *A.meteoricum* differs from *A.albanicum* in having larger bulbs with blackish-brown outer tunics, stem covered up to 1/2 of its length by the leaf sheaths, free spathe valves, with appendage up to 4 mm long, tepals purplish-pink, up to 7.5 mm long, smooth at the apex, staminal filaments longer, whitish, annulus longer, anthers yellow, apiculate at the apex, ovary green, smooth, with much smaller nectariferous pores and smaller capsule. Other relevant differences concern the leaf anatomy, since the leaf cross-section of *A.meteoricum* (cf. Brullo et al. 2001, fig. 11A) is characterised by a thinner cuticle, cells of palisade tissue with uniform size along the entire perimeter, spongy tissue markedly lacunose in the centre and with few vascular bundles in the abaxial face.

According to previous research data (Stearn 1978, Tzanoudakis 1983, Brullo et al. 2001), *A.meteoricum* and *A.albanicum* share the same diploid chromosome complement with 2*n* = 16 and their karyotypes are prevalently constituted by more or less metacentric chromosomes (arm ratio less than 1.67), except for one submetacentric pair, microsatellited in the short arm. The chromosome formulae are also rather similar, with some differences regarding the proportion of *m* and *msm* chromosomes, which are, respectively, 10 and 4 in *A.meteoricum*, contrary to 8 and 6 in *A.albanicum* and the number of recognisable satellited chromosomes, consisting in three pairs of chromosomes for *A.meteoricum* (vs. 4 pairs for *A.albanicum*). The high morphological chromosome homogeneity and karyotype symmetry, rather common in closely allied *Allium* species, accounts for the overall karyological similarity between the two species, with no statistically significant differences in their karyomorphometric parameters (Fig. 3).

Based on literature (Stearn 1978, 1980, Tzanoudakis 1983, Tzanoudakis and Vosa 1988, Brullo et al. 2001), *A.meteoricum* was included in the sect. Scorodon Koch, but as highlighted by Brullo et al. (2018), this traditional section is actually an assemblage of various and well-differentiated phylogenetic lineages (see Fritsch and Friesen 2002, Friesen et al. 2006, Nguyen et al. 2008, Hirschegger et al. 2010, Li et al. 2010).

In particular, the sect. Scorodon s.str., typified by *A.moschatum* L., now belongs to the subgen. Polyprason Radić, which groups rhizomatous species (Friesen et al. 2006, Fritsch et al. 2006b), rather than to subgen. Allium, to which *A.meteoricum* and *A.albanicum* clearly belong. Effectively, there are several species previously included within the sect. Scorodon s.l. which require a taxonomic reassessment, consisting in the recognition of a distinct new section of the subgen. Allium, herein proposed and named as sect. Pseudoscorodon.

### Allium subgen. Allium sect. Pseudoscorodon

Taxon classificationPlantaeAsparagalesAmaryllidaceae

Brullo, C. Brullo, Cambria, Giusso & Salmeri sect. nov.

urn:lsid:ipni.org:names:60478501-2

#### Type.

*Alliumobtusiflorum* DC in Redouté (1805).

#### Diagnosis.

Bulbus solitarius vel bulbilliferous, sine basali rhizomate, folia glabra vel pilosa, numquam filiformes, plerumque spathae valvae umbella breviores, persistentes, saltem 3-nervatae, staminum filamenta complanata inferne, interiores saepe 1–2 cuspidibus praedita, ovarium nectariferis poris bene evolutis, plica membranacea praeditis, partim nectariferum porum tegente.

#### Description.

Bulb solitary or bulbilliferous, leaves glabrous to hairy, never thread-like, spathe valves persistent and usually shorter than the inflorescence, at least 3-nerved, stamen filaments flattened and widened in the lower part, the inner ones often uni-bicuspidate, ovary with well-developed nectariferous pores, bordered by a membranous plica, partly covering the nectariferous pore.

#### Note.

Based on current knowledge (Stearn 1978, 1980, Brullo and Pavone 1983, Pastor and Valdes 1983, Brullo and Tzanoudakis 1989, Tzanoudakis and Kollmann 1991, Brullo et al. 1992a, 1992b, 1993, 1994, 2018, Trigas and Tzanoudakis 2000, Khedim et al. 2016), the following species, all having a Mediterranean distribution, can be included in this new section, in addition to *A.meteoricum* and *A.albanicum*: *A.chalkii* Tzanoud. & Kollmann, *A.chrysonemum* Stearn, *A.erythraeum* Griseb., *A.franciniae* Brullo & Pavone, *A.grosii* Font Quer, *A.lagarophyllum* Brullo, Pavone & Tzanoud., *A.maniaticum* Brullo & Tzanoud., *A.obtusiflorum* DC., *A.reconditum* Pastor, Valdes & Munoz, *A.rhodiacum* Brullo, Pavone & Salmeri, *A.rouyi* G. Gautier, *A.runemarkii* Trigas & Tzanoud., *A.seirotrichum* Ducellier & Maire, *A.thessalicum* Brullo, Pavone, Salmeri & Tzanoud., *A.trichocnemis* Gay and *A.valdecallosum* Maire & Weiller. Amongst these species, we designated as type of the new section *Alliumobtusiflorum*, since it is the oldest known species within this group and a good representative of the new section.

Based on the descriptions and related iconographies, all of these species share the set of discriminant features that characterise the new section and distinguish it very well from all the known sections of the subgenus Allium (Friesen et al. 2006, Khassanov et al. 2011). Altogether, these species markedly differ from *A.moschatum* and consequently from the sect. Scorodon s. str., since the latter shows bulbs with a short basal rhizome, 1–3 mm long (Fritsch et al. 2006b), filiform leaves, spathe valves usually 1-nerved, the larger one rarely obscurely 3-nerved, subulate stamen filaments, ovary with well-developed nectariferous pores which are almost fully covered by a membranous plica.

In order to highlight the morphological similarities and differences amongst the species of the new section, the following analytic key is provided.

### Key to the species referable to the sect. Pseudoscorodon

**Table:** 

1	Leaves hairy	**2**
−	Leaves glabrous or subglabrous	**7**
2	Tepals and stamen filaments greenish-yellow	**3**
−	Tepals and stamen filaments white to pink or purplish	**5**
3	Tepals thickened at the base, 2.5−3.2 mm wide. Inner stamen filaments with 1−2 cusps at the base. Capsule 5−5.5 mm long	*** A. valdecallosum ***
–	Tepals not thickened at the base, 1.5−2.5 mm wide. Stamen filaments all simple. Capsule 3.5−4(4.5) mm long	**4**
4	Leaf blade 0.5−1 mm wide. Umbel fastigiate, 3−4.5 mm long. Stamen filaments exserted	*** A. chrysonemum ***
−	Leaf blade 1.5−2 mm wide. Umbel expanded, 4−6 mm long. Stamen filaments included	*** A. rouyi ***
5	Spathe valves much shorter than umbel. Perigon 4.5−6 mm long. Stamen filaments all simple, exserted	*** A. reconditum ***
−	Spathe valves slightly shorter than umbel (sometimes subequal). Perigon 6−8 mm long. Stamen filaments included, the inner ones with two cusps in the middle part	**6**
6	Leaves almost totally densely hairy. Perigon cup-shaped, white to white-pink with tepals 8−8.2 mm long and 3.2−3.3 mm wide	*** A. seirotrichum ***
−	Leaves sparsely hairy in the sheath. Perigon cylindrical-urceolate, pink-lilac with tepals 5−7 mm long and 1−2 mm wide	*** A. trichocnemis ***
7	Tepals 3.5−5 mm long	**8**
−	Tepals more than 5 mm long	**12**
8	Outer bulb tunics breaking into parallel fibres, pale brown. Inner stamen filaments with two basal cusps	*** A. thessalicum ***
−	Outer bulb tunics coriaceous, brown to dark brown. Inner stamen filaments without basal cusps	**9**
9	Spathe valves free. Umbel with flexuous pedicels	**10**
−	Spathe valves connate at the base. Umbel with erect or suberect pedicels	**11**
10	Leaves 4−6. Inflorescence dense and compact. Spathe valves both 3-nerved, 5−7 mm long. Anthers purple-violet. Ovary with apical purplish-brown spots. Nectariferous pores about ½ of the ovary length	*** A. obtusiflorum ***
−	Leaves 2−4. Inflorescence lax. Spathe valves (1)2−4-nerved, 5−20 mm long. Anthers yellowish. Ovary without apical spots. Nectariferous pores about 1/10^th^ of the ovary length	*** A. maniaticum ***
11	Leaves (3)4−5. Pedicels 2−8 mm long. Tepals whitish-pink. Anthers purplish-violet. Ovary 1.3−2 mm log, with a purplish-brown apical spot	*** A. runemarkii ***
−	Leaves 3. Pedicels 5−20 mm long. Tepals purplish-pink. Anthers pale yellow. Ovary 1.2−1.3 mm long, without apical spot	*** A. erythraeum ***
12	Tepals linear, 5−5.5 × 0.8−1 mm. Ovary 1–1.2 mm long. Capsule max. 3 mm long	*** A. franciniae ***
−	Tepals linear-elliptical to sublanceolate or oblong-elliptical, 5.5−8 × 1.7−2.5 mm. Ovary 1.5−2 mm long. Capsule 3−5 mm long	**13**
13	Spathe valves unilateral, long fused. Inflorescence fastigiate and unilateral	**14**
−	Spathe opposite, free or partially fused. Inflorescence expanded, never unilateral	**15**
14	Stem 15−25 cm long. Inflorescence 12−20-flowered. Tepals purplish at the apex. Ovary 1.6−1.8 mm long	*** A. rhodiacum ***
−	Stem 5−12 cm. Inflorescence 2−12-flowered. Tepals concolorous. Ovary 1.2−1.5 mm long	*** A. chalkii ***
15	Stem flexuous. Spathe valves subequal, fused to half of their length. Tepals white-pink. Ovary with very large nectariferous pores	**16**
−	Stem rigid. Spathe valves unequal, free. Tepals purplish-pink. Ovary with small nectariferous pores	**17**
16	Stem 3-leaved, 14−28(30) cm tall. Spathe valves 8−12 mm long, 3−5-nerved. Style 2.7–2.8 mm long	*** A. albanicum ***
−	Stem 1-leaved, 9−15 cm tall. Spathe valves 5−7 mm long, 1−3-nerved. Style 1 mm long	*** A. lagarophyllum ***
17	Outer bulb coats blackish-brown. Anthers yellow. Staminal annulus 0.7−0.9 mm high. Capsule 3−3.5 mm long	*** A. meteoricum ***
−	Outer bulb coats purplish-brown. Anthers purplish-pink. Staminal annulus 1.5 mm high. Capsule 4−5 mm long	*** A. grosii ***

## Supplementary Material

XML Treatment for Allium albanicum

XML Treatment for Allium subgen. Allium sect. Pseudoscorodon
